# On Some Aspects of Nanobubble-Containing Systems

**DOI:** 10.3390/nano12132175

**Published:** 2022-06-24

**Authors:** Kyuichi Yasui

**Affiliations:** National Institute of Advanced Industrial Science and Technology (AIST), Nagoya 463-8560, Japan; k.yasui@aist.go.jp

**Keywords:** bulk nanobubbles, ultrafine bubbles (UFBs), dynamic equilibrium model, hydrophobic impurity, OH radicals, cavitation, dissolution, rupture of liquid film, surface tension, surface nanobubble

## Abstract

Theoretical studies are reviewed for bulk nanobubbles (ultrafine bubbles (UFBs)), which are gas bubbles smaller than 1 μm in diameter. The dynamic equilibrium model is discussed as a promising model for the stability of a UFB against dissolution; more than half of the surface of a UFB should be covered with hydrophobic material (impurity). OH radicals are produced during hydrodynamic or acoustic cavitation to produce UFBs. After stopping cavitation, OH radicals are generated through chemical reactions of H_2_O_2_ and O_3_ in the liquid water. The possibility of radical generation during the bubble dissolution is also discussed based on numerical simulations. UFBs are concentrated on the liquid surface according to the dynamic equilibrium model. As a result, rupture of liquid film is accelerated by the presence of UFBs, which results in a reduction in “surface tension”, measured by the du Noüy ring method. Finally, the interaction of UFBs with a solid surface is discussed.

## 1. Introduction

Bulk nanobubbles (ultrafine bubbles (UFBs)), which are gas bubbles smaller than 1 μm in diameter, have been commercially applied to cleaning, washing machines, plant cultivation, etc. [1]. In the following, the terminology ultrafine bubble (UFB) is used instead of bulk nanobubble, according to the ISO standardization [2]. However, the details of the mechanisms for the physical, chemical, and biological effects of UFBs are still under debate. Furthermore, the mechanism for stability of UFBs is also still under debate. With regard to the existence of stable UFBs, there have been several experimental reports [3,4,5,6,7,8,9], although there is still skepticism that observed UFBs are not gas bubbles but liquid or solid impurities [10,11]. In a typical production of UFBs, hydrodynamic or acoustic cavitation is used [12]. After stopping cavitation, almost all the microbubbles produced by cavitation disappear at the liquid surface by buoyancy. By contrast, UFBs remain inside the liquid.

Kanematsu et al. [3] experimentally reported that over 90% of the particles that were produced by hydrodynamic cavitation, followed by pulverization by shear force in vortex flow, disappeared after the freeze–thaw process. It may be the case that most of the particles were gas bubbles (UFBs). Other research groups have also reported the disappearance of particles after the freeze–thaw process [4,5]. However, there is a criticism that liquid or solid impurities could also be eliminated from the solution by their aggregation in the freeze–thaw process [11].

Tuziuti et al. [6] experimentally reported that the total volume concentration of the particles, which were produced by the same method as that by Kanematsu et al. [3], decreased as the static pressure increased, while the average size remained nearly constant. It may be the case that most of the particles were gas bubbles (UFBs). However, there is a criticism that liquid impurities could dissolve into water by pressurization [11].

Nirmalkar et al. [4] experimentally reported other pieces of evidence that most of the particles generated after ultrasonic cavitation were gas bubbles (UFBs); their nucleation rate depends strongly on the amount of air dissolved in water, and they gradually disappear with time. Xiao et al. [7] experimentally reported that the number concentration of the particles, which were produced by pressurizing pure nitrogen into water and then slowly depressurizing the solution, decreased suddenly after degassing in a vacuum desiccator. It may be the case that most of the particles were gas bubbles (UFBs). Ke et al. [8] experimentally showed that X-ray fluorescence intensity was correlated with the number density of particles that were produced by pressurizing gas into the solution and then slowly depressurizing it (the compression–decompression method). It suggests that most of the particles had gas inside. Kobayashi et al. [9] experimentally showed by the resonant mass measurement [12] that most of the particles, which were produced by hydrodynamic cavitation using a venturi with pressurization with gas and subsequent depressurization [13], had a density lower than that of liquid water. Furthermore, the diameter of the particles was in a range of 100 to 200 nm, which agrees with the experimental data using other measuring methods, such as nanoparticle tracking analysis [9,12,14]. It may be the case that most of the particles were gas bubbles (UFBs).

In the present paper, theoretical studies of UFBs are reviewed in order to discuss the mechanism of stability, OH radical production, reduction in “surface tension” of the UFB water, and interaction of UFBs with a solid surface.

## 2. Stability

### 2.1. Introduction

It has been experimentally reported that many UFBs are stable in an airtight glass bottle without any gas–liquid interface for more than 9 months [3,15]. There are also other experimental reports on the extreme longevity of UFBs [4,13,16,17,18,19]. It is a mystery because a gas bubble of 100 to 200 nm in diameter should completely dissolve into liquid water in less than 1 ms according to the Epstein–Plesset theory [20,21]. In order to explain the mystery, several theoretical models have been proposed, such as charge-stabilization model [22,23,24,25,26,27,28], dynamic equilibrium model [29], high inner-density model [30,31], etc. A charge-stabilization model has been discussed most frequently; a negatively charged UFB is stabilized against dissolution by electrostatic repulsive force, which may compensate the Laplace pressure due to surface tension. Some calculations have shown, however, that the ratio of electrostatic pressure to Laplace pressure is much less than $10^{-2}$, which suggests that the electrostatic interaction may not be the main factor for stabilizing UFBs [32]. Furthermore, the reduction in surface tension of UFB water observed experimentally may not be explained by a charge-stabilization model because UFBs are expected to burst and disappear at the liquid surface according to the model [33,34,35]. In the present section, a dynamic equilibrium model is discussed because there is experimental evidence of the TEM (Transmission Electron Microscopy) observation, as well as the fact that UFBs do not necessarily disappear at the liquid surface according to the model [16,17,36,37]. With regard to the high inner-density model that the lifetime of UFBs is longer as the density inside a bubble is higher, experimental evidence is required [38]. As the existence of the boundary layer of a UFB has been confirmed experimentally [39], its role on stability of a UFB should be studied further [40,41]. Other models for the stability of a UFB are discussed in Refs. [36,42].

### 2.2. Dynamic Equilibrium Model

In the present subsection, a dynamic equilibrium model for the stability of a UFB is discussed. The dynamic equilibrium model was first proposed by Brenner and Lohse [43] as a model for a surface nanobubble. A surface nanobubble is a gas object on a solid surface in liquid water with a footprint diameter ranging from 50 nm to 2 μm and a height ranging from 10 to 100 nm [44,45,46]. Brenner and Lohse [43] proposed that a surface nanobubble is stabilized against dissolution by the gas influx near the contact line on a hydrophobic surface where the gas is attracted. The author and his coworkers [47] have extended the model to a surface nanobubble on a hydrophilic surface by taking into account the van der Waals attraction between gas molecules inside a surface nanobubble and the solid surface. According to the model [47], pressure inside a surface nanobubble depends on the distance from the solid surface. Accordingly, the shape of the micropancake, which is a nearly-two-dimensional bubble, is reproduced by the model due to the strong dependence of the radius of curvature on the distance from the solid surface [47]. It is also shown by numerical calculations that a surface nanobubble could be stable, even in liquid water undersaturated with gas [47].

**Figure 1:**
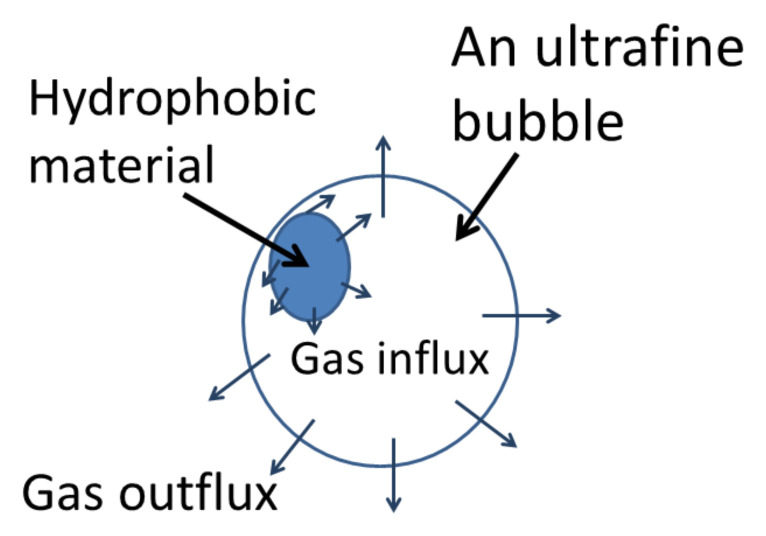
Dynamic equilibrium model [29]. Copyright (2016), with permission from American Chemical Society.

The author and his coworkers [29] applied the model to a UFB partly covered with a hydrophobic impurity (Figure 1). We assume that a hydrophobic impurity could be carbon particles, oils, etc., mainly produced from a UFB generator [48]. As a hydrophobic material repels liquid water, there is a density depletion layer of 0.2–5 nm in thickness on a hydrophobic surface, in which water density is decreased to 44–94% [49,50]. These have been experimentally measured with X-ray or neutron reflectometry [49,50]. As a result, gas is preferentially trapped in the depletion layer [51,52,53,54,55]. The gas pressure near the surface of a hydrophobic material ($p_{dis}$) is crudely estimated as follows.

(1) $$p_{dis}=p_{dis,\infty }e^{-\frac{\Phi }{k_{B}T}}$$

where $p_{dis,\infty }$ is the pressure of gas dissolved in the liquid far from a hydrophobic surface, Φ is the potential of hydrophobic attraction ($\Phi =-1.7\times 10^{-20}$ J) [47,56,57], $k_{B}$ is the Boltzmann constant (=$\;1.38\times 10^{-23}$ J/K), and T is temperature in K. According to Equation (1), the gas pressure near the hydrophobic surface is about 67 atm when the pressure of gas dissolved in the liquid far from a hydrophobic surface is 1 atm at 20 °C (293 K).

**Figure 2:**
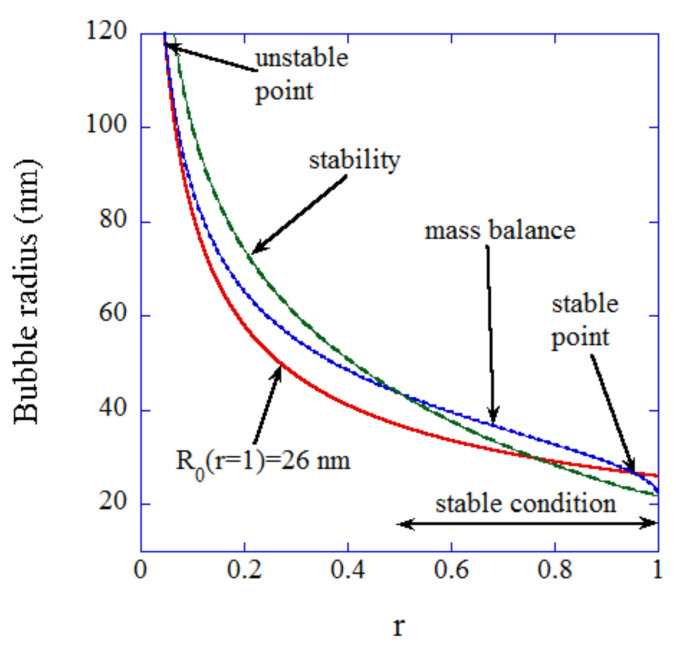
Mass balance condition (blue dotted line) and the stability threshold curve (green dashed line) as a function of the fraction of surface coverage (r) by a piece of hydrophobic material [29]. Above the stability threshold curve, the mass balance condition is stable. The red solid line is the bubble radius when the surface area of the hydrophobic material is kept constant. Copyright (2016), with permission from American Chemical Society.

When the radius of a UFB is 100 nm, the internal gas pressure of a UFB is about 15 atm. When a part of the UFB surface is covered with a hydrophobic material (Figure 1), gas diffuses into a bubble from the periphery of a hydrophobic material on a bubble surface because the gas pressure at the hydrophobic surface (67 atm) is higher than the internal gas pressure (15 atm). On the other hand, gas diffuses out of a bubble from the other uncovered surface of a bubble because the internal gas pressure (15 atm) is higher than the gas pressure dissolved in the liquid (1 atm). When the gas influx and outflux are balanced, a UFB is stabilized against dissolution. In addition, this balance should be stable, such that a slight change in bubble radius results in the return to the initial equilibrium radius, which is called stable equilibrium. The stable condition is above the green dashed line in Figure 2. The mass balance condition is shown by the blue dotted line. The details of the equations are described in Ref. [29]. Thus, the stable equilibrium condition is the blue dotted line above the green dashed line in Figure 2. In other words, when the fraction of surface coverage by a hydrophobic material is more than about 50%, a UFB could be stable against dissolution.

**Figure 3:**
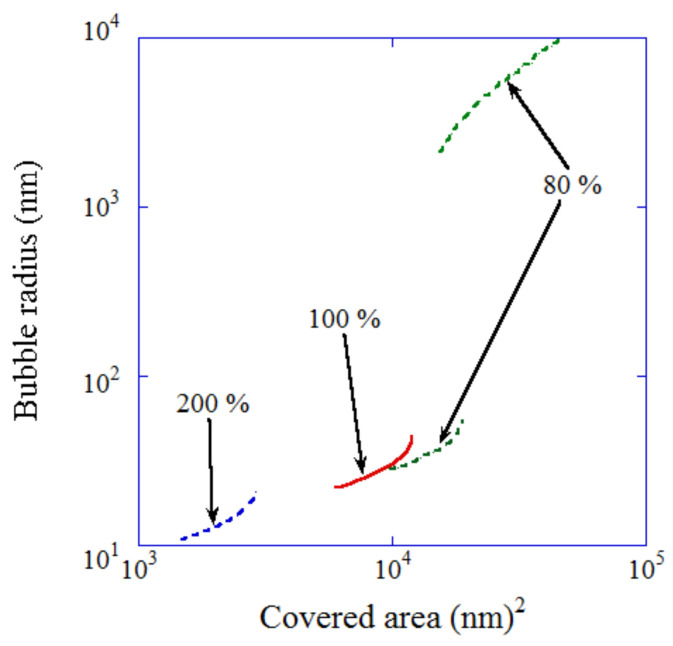
Stable bubble radius as a function of the area covered with a piece of hydrophobic material for various degrees of gas saturation [29]. Copyright (2016), with permission from American Chemical Society.

According to the dynamic equilibrium model, the stable conditions for a UFB are only for a restricted range of surface area covered with a hydrophobic impurity, as shown in Figure 3. The range of stable bubble radius is 22–44 nm for gas-saturated water, 28–55 nm in slightly degassed water with 80% gas saturation, and 11–21 nm for supersaturated water with 200% gas saturation (Figure 3). For the slightly degassed water, a microbubble with a radius larger than 2.1 μm is also stabilized with the fraction of surface coverage smaller than $3\times 10^{-4}$ (Figure 3). Such microbubbles would be the cavitation nuclei reported many years ago [58,59,60,61]. Although the theoretical estimate of the tensile strength of pure water is in the order of 1000 atm, the actual threshold pressure amplitude of ultrasound for cavitation to occur is as small as ~1 atm [60,61,62,63]. Acoustic cavitation is the formation of bubbles that subsequently collapse in liquid irradiated by strong ultrasound [60,61,64]. The discrepancy between the theoretical tensile strength and the actual cavitation threshold is due to the presence of cavitation nuclei in the actual experiments. The cavitation nuclei are solid impurities or stabilized microbubbles or UFBs [58,59,60,61,65,66,67]. Solid impurities work as cavitation nuclei because bubbles are more easily nucleated at the crevices of such particles [65,66,67]. According to Figure 3, stable microbubbles partly covered with hydrophobic impurities could be the origin of the cavitation nuclei.

Nevertheless, the dynamic equilibrium model has been criticized due to the following two reasons. One is that the permanent gas circulation in the model may be a perpetual motion machine. In other words, the model may violate the laws of thermodynamics. The other is that liquid flow was not experimentally detected around a surface nanobubble [68,69]. However, the liquid flow is not assumed in the model because only diffusion of gas in quiescent liquid is assumed. With regard to the former problem, the change in energy and entropy at each process of dissolution and diffusion is analytically calculated in Ref. [29]. As a result, the energy is conserved and the total change in entropy is zero [29]. The total entropy change could be zero only when the state is in equilibrium, which satisfies the second law of thermodynamics [70]. In other words, the model satisfies the first and second laws of thermodynamics, and the permanent gas circulation in the model is not a perpetual motion machine.

Sugano, Miyoshi, and Inazato [16,17] reported the TEM images of UFBs partly covered with hydrophobic materials in aqueous solution without freezing. The UFB water was introduced into the MEMS (Microelectromechanical System) chip to make a very thin liquid layer of several hundred nm. The MEMS chip was mounted on an in-situ holder of TEM. The hydrophobic materials were oleic-acid, α-tocopherol, etc., which were added in the aqueous solutions. The experimental observation of stable UFBs partly covered with hydrophobic materials would be the experimental evidence of the dynamic equilibrium model. For the TEM images of UFBs, please see References [16,17,36].

## 3. Generation of OH Radicals

### 3.1. Introduction

There are several experimental reports that OH radicals are produced from UFBs [71,72,73,74,75]. Liu et al. [71] reported that OH radicals were detected from UFB water using a sensitive fluorescence probe, APF. The fluorescence intensities were measured for UFB water in which APF was added. UFB water was produced by introducing pure oxygen into a phosphate buffer and by circulating the liquid through a UFB generator [71]. The increase in the fluorescence intensity by the addition of Fe^2+^ was considerably lower than that expected if all the reactive oxygen species (ROS) were H_2_O_2_. Thus, the ROS in the UFB water were mostly identified as OH radicals. Takahashi et al. [72] experimentally reported that OH radicals were detected by the ESR measurement from ozone UFB water, which was produced by the pressurized dissolution method and kept in plastic bottles in a dark place at room temperature for approximately six months. Jin et al. [75] experimentally reported that OH radicals were detected using the fluorescence probe APF near the periphery of a liquid film of UFB water, which was produced by the pressurized dissolution and decompression method using a piston [5]. There is also a negative experimental report on OH production from UFB water [76].

In relation to the generation of OH radicals from UFB water, the generation of OH radicals from microbubble water has also been experimentally reported [77,78]. Wang et al. [77] reported that OH radicals were detected using the fluorescence probe APF from microbubble water during microbubble generation using a microbubble generator, consisting of high-speed rotation and compression–dissolution processes. OH radicals were also detected after stopping the microbubble generation for about 20 min [77]. Arrojo et al. [78] experimentally reported that OH radicals were detected by circulating 30 L of a 250 ppm salicylic acid solution through a cavitation loop with a Venturi tube. Salicylic acid reacts with OH radicals and the products were detected by using HPLC (High-Performance Liquid Chromatographer). The OH radical yield increased as the number of the circulation cycles increased up to 6 μM after 250 cycles [78].

In the present section, the mechanism of OH radical production from UFB water is discussed based on the results of numerical simulations. Firstly, the mechanism of OH radical production during acoustic or hydrodynamic cavitation to produce UFBs is discussed. Next, the possibility of OH radical production from dissolving UFBs is discussed. At last, the mechanism of OH radical production after stopping cavitation is discussed.

### 3.2. OH Production during Cavitation

**Figure 4:**
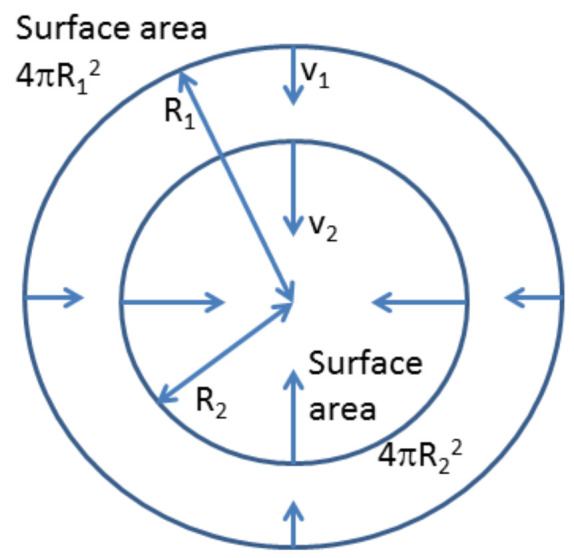
Spherically inward flow as the mechanism for the violent collapse of a bubble [21]. Copyright (2015), with permission from Elsevier.

Since 1982, it has been widely known that OH radicals are generated during acoustic or hydrodynamic cavitation [64,79]. In acoustic or hydrodynamic cavitation, a cavitation bubble of several micrometers in ambient radius, which is the bubble radius when ultrasound or pressure disturbance is absent, violently collapses [80]. There are two reasons for the violent bubble collapse [21,64]. One is the spherical geometry of a collapsing bubble (Figure 4). According to the continuity of the liquid, the speed of the inflowing liquid increases as the distance from the center of the sphere (bubble center) decreases because the surface area decreases. The other is the inertia of the inflowing liquid. The violent bubble collapse is called the Rayleigh collapse [64,81]. At the end of the violent bubble collapse, temperature and pressure inside a bubble significantly increase to several thousand Kelvin and several hundred atmospheric pressure or more, respectively [82,83]. As a result, OH radicals are produced inside a bubble by thermal dissociation of water vapor molecules, as well as oxygen molecules, if present [84,85]. In addition, O radicals, H_2_O_2_, and O_3_ are produced inside a bubble, which are oxidants [84]. H_2_, H, HNO_2_, HNO_3_, NO, and HO_2_ are also produced inside an air bubble [84]. There are also a few experimental reports on the production of NH_3_ [64,86,87]. Due to the high temperature and pressure inside a bubble, gases inside a bubble are weakly ionized, partly due to the ionization potential lowering by the high density inside a bubble [80,88,89,90,91]. As a result, a faint light is emitted from a bubble at the violent bubble collapse as a pulse with a continuum optical spectrum due to emissions from plasma and OH-line at 310 nm in wavelength due to chemiluminescence, which is called sonoluminescence (SL) [88,92]. From the optical spectra of SL, the temperature and pressure inside a bubble can be measured [82,83,93].

**Figure 5:**
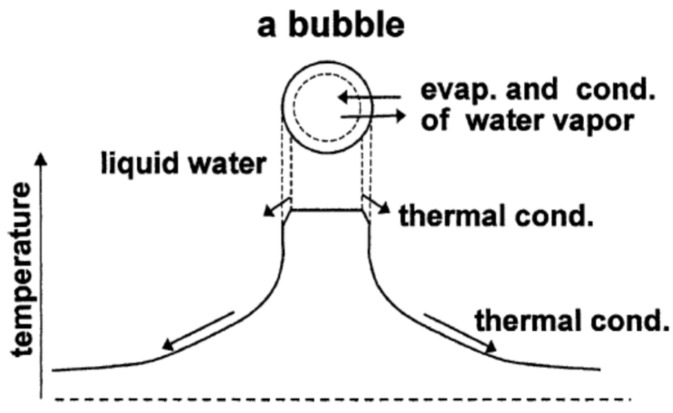
The model of bubble dynamics [103]. Copyright (2004), with permission from Elsevier.

Single-bubble sonoluminescence (SBSL) is the light emission from a single stable bubble trapped near the pressure antinode of a standing ultrasonic wave [81,92,94]. SL from many cavitation bubbles is called multibubble sonoluminescence (MBSL) [88,92]. The single-bubble system is much more suitable for direct comparison between theory and experiment because the acoustic pressure at the position of the bubble can be measured experimentally. In addition, there is no bubble–bubble interaction [64,95,96,97]. In 2002, Didenko and Suslick [98] experimentally reported in *Nature* that the number of OH radicals produced from the single-bubble system was $8.2\times 10^{5}$ per acoustic cycle at the liquid temperature of 3 °C. We performed numerical simulations of OH radical production from the single-bubble system under the experimental condition [99]. The bubble dynamics model has been developed through the study of SBSL [64,99,100,101,102,103,104]. In the model, temperature and pressure are assumed to be spatially uniform inside a bubble except at the thermal boundary layer near the bubble wall (Figure 5). In the model, the following effects have been taken into account: non-equilibrium evaporation and condensation of water vapor at the bubble wall, thermal conduction both inside and outside the bubble, variation in liquid temperature at the bubble wall, non-equilibrium chemical reactions inside the bubble, and ionization of gases inside the bubble with ionization potential lowering due to the high density inside a bubble. With regard to chemical reactions inside an air bubble, rates of chemical reactions for 93 chemical reactions and their backward reactions are numerically calculated. Details of the chemical kinetics model is described in Ref. [105]. It should be noted, however, that recently, Kalmár et al. [106] pointed out that the results of numerical simulations on the amounts of chemical products, such as OH radicals, strongly depend on the chemical kinetics model used in the simulations.

**Figure 6:**
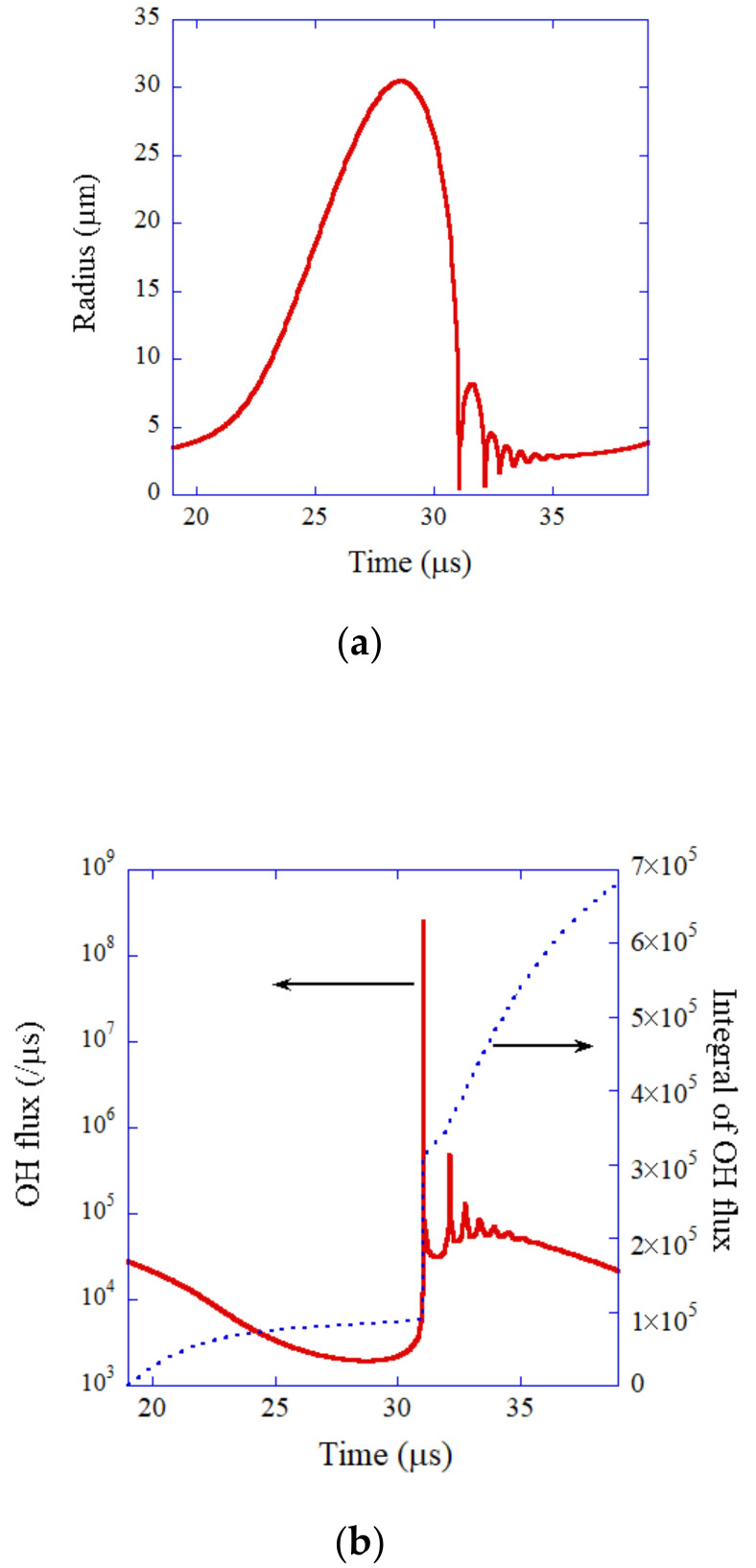
The results of numerical simulation for one acoustic cycle when an SBSL bubble in a steady state in water is irradiated by an ultrasonic wave of 52 kHz and 1.52 bar in frequency and pressure amplitude, respectively [99]. The ambient bubble radius is 3.6 μm. (**a**) The bubble radius. (**b**) The dissolution rate of OH radicals into the liquid from the interior of the bubble (red solid line) and its time integral (blue dotted line). Copyright (2005), with permission from AIP Publishing.

In the single-bubble system, nitrogen and oxygen in an air bubble chemically react to form NOx and HNOx inside the heated bubble at each violent collapse [107,108]. In the single-bubble system, a bubble repeats expansion and collapse in a clock-like manner [109,110]. As a result, the amount of nitrogen and oxygen inside a bubble gradually decreases because NOx and HNOx gradually dissolve into the surrounding liquid water. Finally, the content of the bubble becomes mostly argon because 1% of air is argon in the molar fraction. This argon rectification hypothesis has been validated both theoretically and experimentally [81,107,108]. Thus, the results of numerical simulations for an argon bubble are shown in Figure 6 under the condition of the single-bubble experiment [98,99].

During the rarefaction phase of ultrasound, a bubble considerably expands (Figure 6a). At the compression phase of ultrasound, a bubble violently collapses followed by small bouncing motions. The OH flux from the interior of a bubble into the surrounding liquid takes a maximum value at the violent bubble collapse (Figure 6b). About 1/3 of the total amount of OH radicals dissolving into the surrounding liquid in one acoustic cycle dissolves at the violent bubble collapse. For the other 2/3, OH radicals gradually dissolve into the surrounding liquid from the interior of the bubble during bubble expansion and bouncing motions. The total number of OH radicals dissolving into the surrounding liquid per acoustic cycle is $6.6\times 10^{5}$, which almost agrees with the experimental data of $8.2\times 10^{5}$ [98,99]. Thus, the present model has been validated.

**Figure 7:**
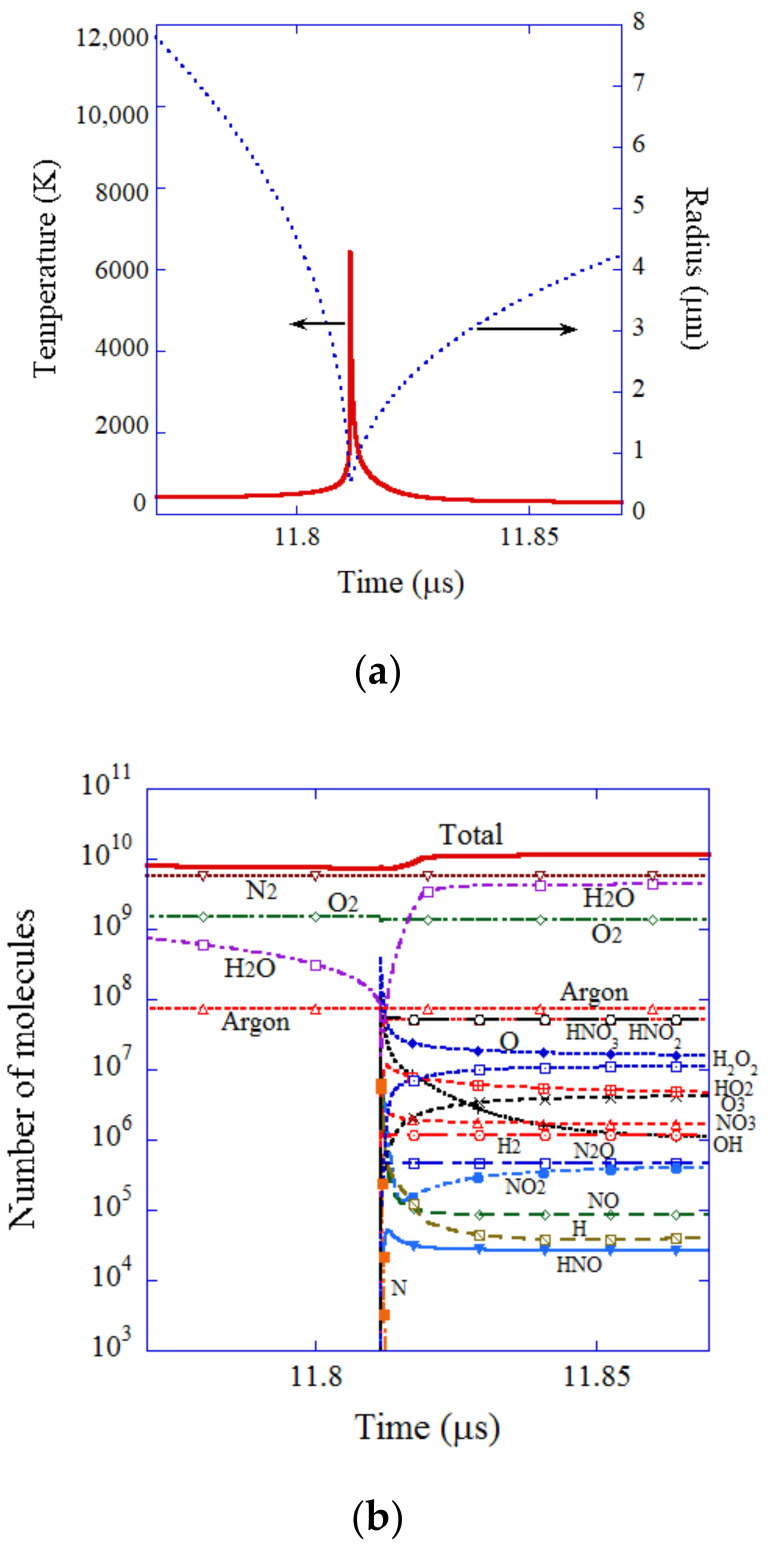
The results of numerical simulation for an initial air bubble at the end of the bubble collapse only for 0.1 μs [99]. (**a**) The bubble radius. (**b**) The number of molecules inside a bubble. Copyright (2005), with permission from AIP Publishing.

The results of the numerical simulations for an initial air bubble are shown in Figure 7 as a function of time at the end of the violent collapse [99]. In Figure 7a, the bubble radius is shown by a blue dotted line and the temperature inside the bubble is shown by a red solid line. The bubble violently collapses, and the bubble temperature sharply increases to 6500 K at the end of the violent collapse. Then, the bubble immediately expands again, and the bubble temperature sharply decreases. Due to the high temperature at the end of the violent collapse, most of the water vapor molecules are dissociated inside the bubble (Figure 7b). Furthermore, many of the oxygen and nitrogen molecules chemically react and many chemical products are produced, such as HNO_3_, HNO_2_, O, H_2_O_2_, HO_2_, O_3_, NO_3_, OH, H_2_, N_2_O, NO_2_, etc. The amount of the chemical products that dissolve into the liquid from the interior of an initial air bubble in one acoustic cycle is as follows, in descending order, according to the numerical simulation [99]: HNO_2_: $4.0\times 10^{7}$, HNO_3_: $3.7\times 10^{7}$, O: $1.6\times 10^{7}$, H_2_O_2_: $5.1\times 10^{6}$, O_3_: $2.7\times 10^{6}$, HO_2_: $2.3\times 10^{6}$, NO_3_: $1.1\times 10^{6}$, H_2_: $1.0\times 10^{6}$, OH: $9.9\times 10^{5}$, etc. In summary, appreciable amounts of OH radicals, H_2_O_2_, and O_3_ molecules are produced from an air bubble under acoustic cavitation. It is also true for hydrodynamic cavitation, such as being used widely in the production processes of UFBs, because the violent bubble collapse (the Rayleigh collapse) in hydrodynamic cavitation is similar to that in acoustic cavitation, as already noted [91,111,112,113].

### 3.3. Radical Production during Bubble Dissolution

In many of the experiments [71,72,73,74,75,77], OH radicals were detected after stopping cavitation. In the absence of solutes, the lifetime of OH radicals in liquid water is determined by the following reaction.

(2) $$OH+OH\;\to \;H_{2}O_{2}$$

According to Henglein [85], the local concentration of OH radicals that reach the liquid phase from the interior of a cavitation bubble is experimentally estimated as $5\times 10^{-3}$ M. As the rate constant for reaction (2) at room temperature is $1\times 10^{10}$ M^−1^ s^−1^ [114], the lifetime of OH radicals around a cavitation bubble is estimated as 20 ns [64]. Accordingly, OH radicals detected after stopping cavitation [71,72,73,74,75,77] should not be produced during cavitation but produced after stopping cavitation. In the present subsection, the possibility of OH radical production during dissolution of a bubble is discussed based on the results of numerical simulations [115,116,117].

**Figure 8:**
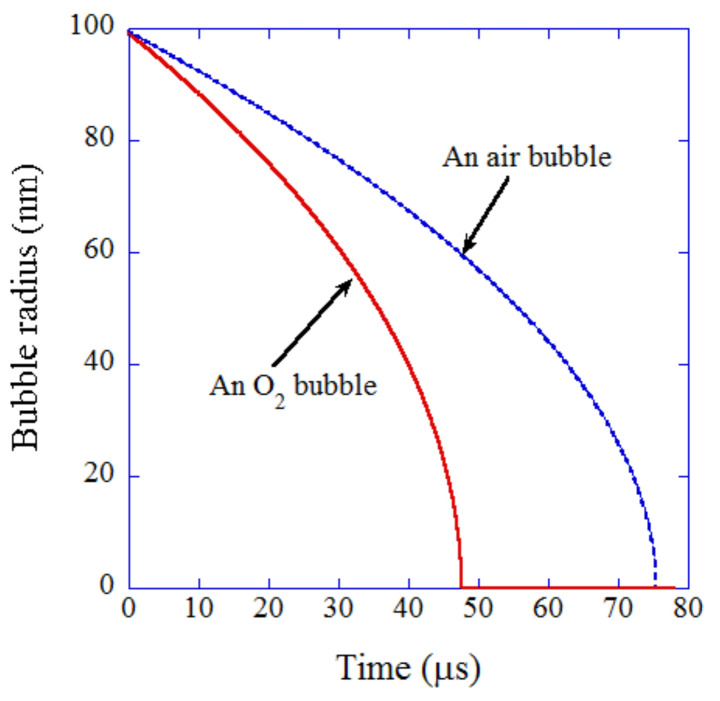
The results of numerical simulation for dissolution of an oxygen or air UFB into gas-saturated water [115]. The bubble radius as a function of time with an initial value of 100 nm. Copyright (2019), with permission from Elsevier.

In the numerical simulations of bubble dissolution [115,116,117], an equation of gas diffusion rate is coupled with the bubble dynamics model, validated from the study of the single-bubble system described above. The initial bubble radius is assumed as 100 nm, which is typical for UFBs. An oxygen bubble dissolves faster than an air bubble (Figure 8) [115]. The time for the complete dissolution of an oxygen bubble is about 47.6 μs, while that for an air bubble is 75.4 μs according to the numerical simulations [115,116]. According to the Epstein–Plesset theory [20,21], in which the effect of the bubble dynamics (the inertia of the surrounding liquid) is neglected, they are 48.3 μs and 77.8 μs for oxygen and air bubbles, respectively, which nearly agree with the above results.

**Figure 9:**
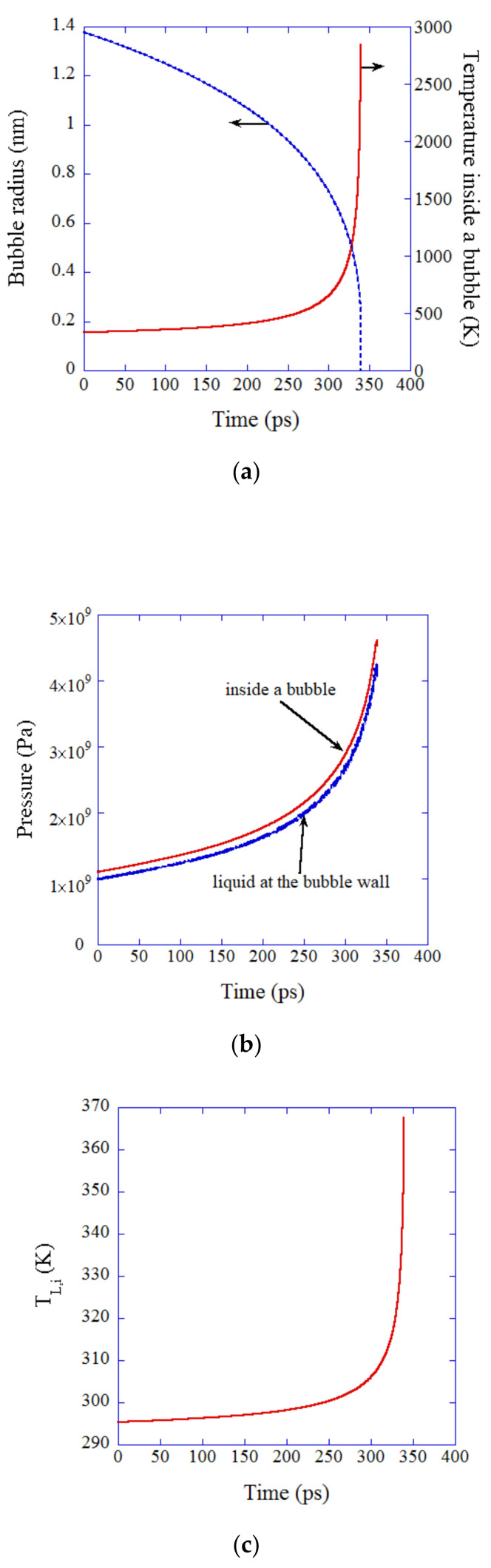

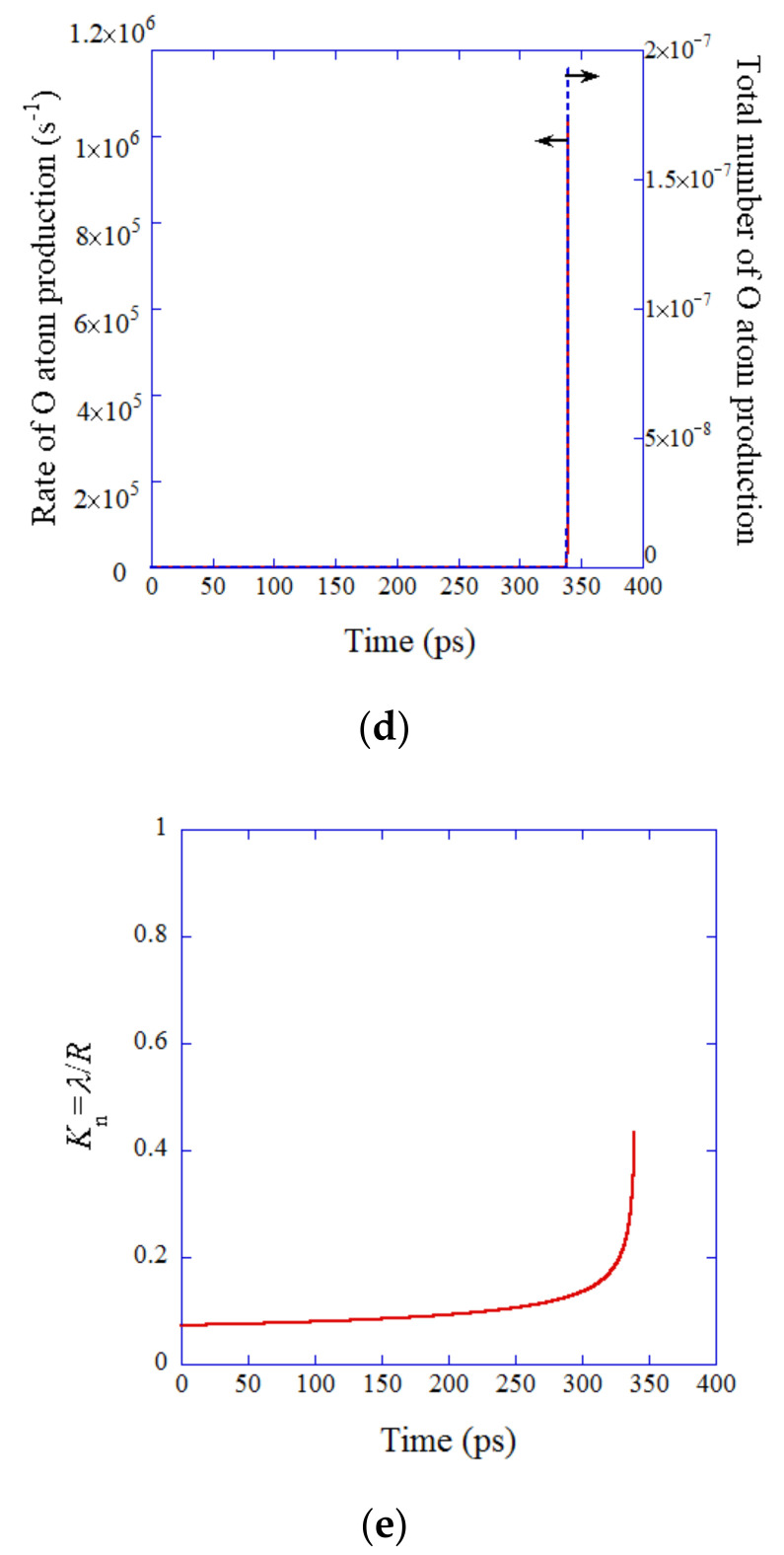
The results of the numerical simulation as a function of time for the last 340 ps of the complete dissolution of an oxygen UFB into water saturated with oxygen [115]. The condition is the same as that in Figure 8 (t = 0 in this figure corresponds to t = 47.579 μs in Figure 8). (**a**) The bubble radius (blue dotted line) and the temperature inside a bubble (red solid line). (**b**) The pressure inside a bubble (red solid line) and the liquid pressure at the bubble wall (blue dotted line). (**c**) The liquid temperature at the bubble wall. (**d**) The rate of O atom production (red solid line) and the total number of O atoms produced (blue dotted line). (**e**) The Knudsen number. Copyright (2019), with permission from Elsevier.

The results of the numerical simulation near the final moment of the complete dissolution of an oxygen bubble are shown in Figure 9 [115]. Surprisingly, the temperature inside a bubble increases to 2800 K at the final moment of the complete dissolution (Figure 9a). The reason is similar to that of the Rayleigh collapse in cavitation; *pV* work done on a bubble by the surrounding liquid overwhelms the energy loss due to thermal conduction from the heated interior of a bubble to the surrounding liquid [115].

The pressure inside and outside a bubble increases to about 4.5 GPa and 4 GPa, respectively, at the final moment of the bubble dissolution (Figure 9b). This is also similar to the Rayleigh collapse in cavitation. The liquid temperature at the bubble wall increases to about 94 °C due to the thermal conduction from the interior of the heated bubble (Figure 9c). The liquid temperature of 94 °C is insufficient for the thermal dissociation of water molecules. Thus, the only possible radical formation is the dissociation of O_2_ molecules inside a bubble. According to the numerical calculations of the rate of O_2_ dissociation, the number of O atoms produced inside a dissolving oxygen bubble is in the order of $10^{-7}$ (Figure 9d). In other words, only a few molecules of O radicals could be formed per $10^{7}$ dissolving oxygen bubbles. It means that the OH radicals detected in the experiments [71,72,73,74,75,77,78] could not be originated from dissolving bubbles. It should be noted, however, that the accuracy of the present numerical simulations is not high because the Knudsen number, which is the instantaneous mean free path of a gas molecule inside a bubble divided by the instantaneous bubble radius, becomes considerably larger than 0.1 (Figure 9e). It means that the continuum model assumed in the present numerical simulations is no longer valid, and molecular dynamics simulations should be performed under the conditions.

Takahashi et al. [72,73,74] suggested that OH radicals may be generated by the accumulation of electric charges near the bubble surface at the final moment of the complete bubble dissolution. They experimentally suggested that zeta potential of a dissolving UFB increases as the bubble shrinks. As a result, the electric charge density near the bubble surface significantly increases at the final moment of the complete dissolution of the bubble. It is suggested that due to the extreme accumulation of the electric charges near the bubble surface, OH radicals may be generated. This possibility should be studied in future.

### 3.4. Radical Production by Chemical Reactions in Liquid

In the previous subsection, the results of the numerical simulations suggest that OH radicals detected in the experiments after stopping cavitation [71,72,73,74,75,77] are not originated from dissolving bubbles. Then, what is the origin of the OH radicals generated after stopping cavitation? One possibility is the following chemical reactions of H_2_O_2_ and O_3_ in the liquid, which are produced during hydrodynamic or acoustic cavitation, to produce UFBs [117].

When 5 < pH < 8,

(3) $$H_{2}O_{2}\to HO_{2}^{-}+H^{+}$$

(4) $$O_{3}+HO_{2}^{-}\to OH+O_{2}^{-}+O_{2}$$

When pH < 5,

(5) $$H_{2}O_{2}+O_{3}\to OH+HO_{2}+O_{2}$$

The reaction mechanisms (3)–(5) are based on the experimental observation of the reaction between O_3_ and H_2_O_2_ in liquid water [118,119]. Reaction (5) is relatively slow [118].

The reaction of O_3_ and OH^-^ also produces OH radicals as follows [118,120,121,122].

When pH > 8,

(6) $$O_{3}+OH^{-}\to HO_{2}+O_{2}^{-}$$

(7) $$O_{2}^{-}+O_{3}+H^{+}\to OH+2O_{2}$$

Takahashi et al. [72] experimentally reported that OH radicals were detected under strong acidic conditions. In their experiment [72], FeSO_4_ was added to water. During the generation of ozone microbubbles as well as UFBs by pressurizing gas into the solution followed by depressurization (hydrodynamic cavitation), precipitates of Fe_2_O_3_ and FeO may be formed through the reactions with oxidants, such as O_3_ and H_2_O_2_. By the addition of strong acid (HCl), $Fe^{2+}$ ions may be formed again by the dissolution of the precipitate (FeO), and the following Fenton reaction [122] may occur with H_2_O_2_, created by hydrodynamic cavitation.

(8) $$Fe^{2+}+H_{2}O_{2}\to Fe^{3+}+OH+OH^{-}$$

Another possibility is OH radical production through the dissociation of O_3_ if some amount of O_3_ still remained in the solution after six months in a dark place at room temperature. Further studies are required on this topic.

## 4. Surface Tension of UFB Water

**Figure 10:**
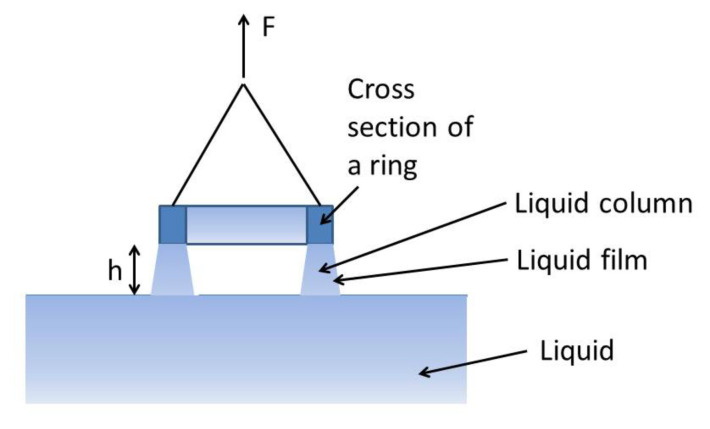
The du Noüy ring method for surface tension measurement [37]. Copyright (2019), with permission from Elsevier.

Ushida et al. [33] experimentally reported that the surface tension of UFB water measured by the du Noüy ring method was about 64 mN/m, which is about 10% lower than that of pure water of about 73 mN/m. In the du Noüy ring method, surface tension (σ) is measured by the force balance equation (Equation (9)) when the liquid film is ruptured as the ring is moved upward (Figure 10) [33,37].

(9) $$F=2\pi \left(r_{1}+r_{2}\right)\sigma +\pi \left(r_{1}^{2}-r_{2}^{2}\right)\rho_{L}gh$$

where F is the maximum value of the force in Figure 10, $r_{1}$ and $r_{2}$ are outer and inner radii of the ring, $\rho_{L}$ is the liquid density, and h is the height of the ring when the liquid film is ruptured in Figure 10. In other words, the surface tension is determined by the height (h) of the ring when the liquid film is ruptured.

It has been experimentally reported that the surface tension of liquid water is considerably reduced when the liquid surface is almost filled with hydrophobic solid particles [123,124,125,126]. The surface concentration of hydrophobic particles (polymeric particles) for the considerable reduction in surface tension is in the order of $10^{9}$ cm^−2^ [123].

**Figure 11:**
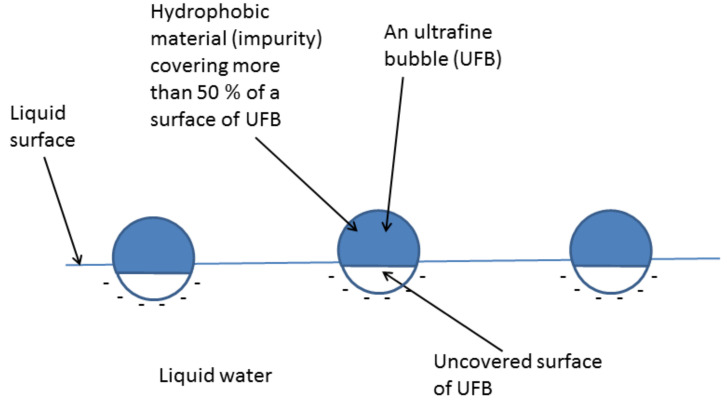
UFBs concentrated on a surface of liquid water [37]. Each UFB is partially covered with a hydrophobic material. Copyright (2019), with permission from Elsevier.

According to the dynamic equilibrium model [29] of UFBs discussed in Section 2, more than half of the surface of a UFB is covered with a hydrophobic material. As the free energy of a hydrophobic material is lower above the liquid surface in the gas phase than that inside liquid water, some UFBs are expected to be concentrated at the liquid surface by directing the hydrophobic cap upward (Figure 11) [37]. If the surface of a UFB is not covered with a hydrophobic material, a bubble immediately bursts and disappears at the liquid surface. When more than half of the UFB surface is covered with a hydrophobic material, some part is underneath the liquid surface due to the gravitational force (Figure 11). As a result, a UFB does not burst, even at the liquid surface.

Then, could the liquid surface be almost filled with UFBs enough to reduce surface tension of the liquid? The surface concentration of UFBs at the water surface is estimated by the change in the Gibbs free energy of a UFB at the liquid surface. The change in the Gibbs free energy is mainly given by the decrease due to escape of the hydrophobic material from liquid water as well as the decrease due to the reduction in solid–liquid and liquid–gas interface areas, subtracted by the increase due to solid–gas interface area [37]. Assuming a UFB concentration in the bulk liquid of $10^{8}$ cm^−3^, the surface concentration of UFBs at the water surface is estimated as in the order of $10^{5}$ cm^−2^ or less [37]. This surface concentration is about 4 orders of magnitude smaller than that required for a reduction in surface tension by hydrophobic particles in the order of $10^{9}$ cm^−2^ [123]. In other words, the surface concentration of UFBs at the water surface is too small to reduce the surface tension of water.

Then, what is the reason for the reduction in “surface tension” by about 10% measured by the ring method for UFB water? One possibility is that rupture of the liquid film is accelerated by the presence of UFBs at the liquid surface. When the uncovered part of the surface of a UFB is directed upward by some disturbance, the bubble bursts and disappears at the liquid surface like a normal bubble. At the moment, the liquid film, such as that in Figure 10, may be ruptured. Then, the value of “surface tension” measured by the du Noüy ring method decreases according to Equation (9). This possibility should be studied further, both theoretically and experimentally.

## 5. Interaction with a Solid Surface

As discussed in Section 2.1, it has been experimentally reported that UFBs are stable for more than a month after their generation [3,4,13,15,16,17,18,19]. There are three aspects in their stability [127]. One is the diffusive stability that a UFB is stable against dissolution into the liquid, discussed in Section 2. Another is colloidal stability against their aggregation. The final aspect is stability against adsorption on a container’s wall (solid surface). In the present section, stability against adsorption on a solid surface is discussed based on theoretical analysis [127] of the experimental results of Kanematsu et al. [3].

In the experiment of Kanematsu et al. [3], the temporal change in number concentration of UFBs was measured using the nanoparticle tracking analysis (NTA) [12]. UFBs were generated using a commercially available bubble generator, in which hydrodynamic cavitation followed by pulverization by shear force in vortex flow was utilized. Most of the produced particles were confirmed as UFBs by the decrease in the number concentration, more than 90% after the freeze–thaw process [3]. Three kinds of polymer materials were immersed in the UFB water in a glass bottle with a screw-on cap without a gas–liquid boundary at 25 °C. The polymer materials tested were polyethylene (PE) tubes, nylon balls made of nylon 66, and poly(ethylene terephthalate) (PET) stripes [3]. The contact area against each polymer material per unit volume of the solution was adjusted to about 2.88 cm^2^/mL. The initial number concentration of UFBs ranged from $1.15\times 10^{9}$ to $1.58\times 10^{9}$ mL^−1^, which was achieved by the circulations of the water going through the generator 120 times [3]. UFBs had diameters ranging from 40 to 600 nm, and the typical diameter was 70 nm. The UFB concentrations decreased with time, particularly for the first 5 days, followed by a slower decrease [3]. The decreasing number concentrations exposed to PET and PE leveled off after about 10 days. For nylon balls, the UFB concentration continued decreasing, even after 25 days. Just after 28 days, the decrease in the UFB concentration was about 30% of the initial value for nylon balls, about 18% for PE, and only about 2% for PET [3].

**Figure 12:**
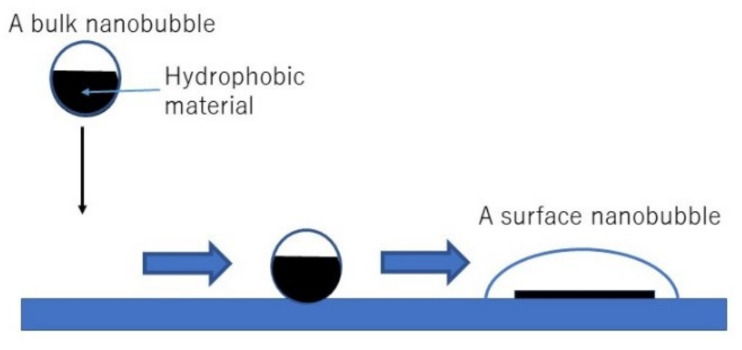
Interaction of UFB with a solid surface [127]. Copyright (2021), with permission from American Chemical Society.

**Figure 13:**
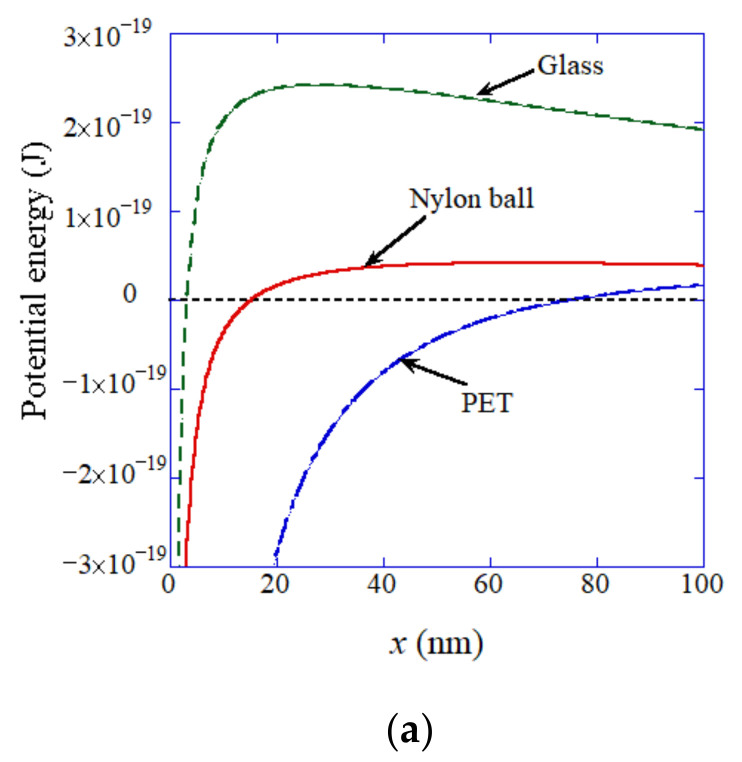

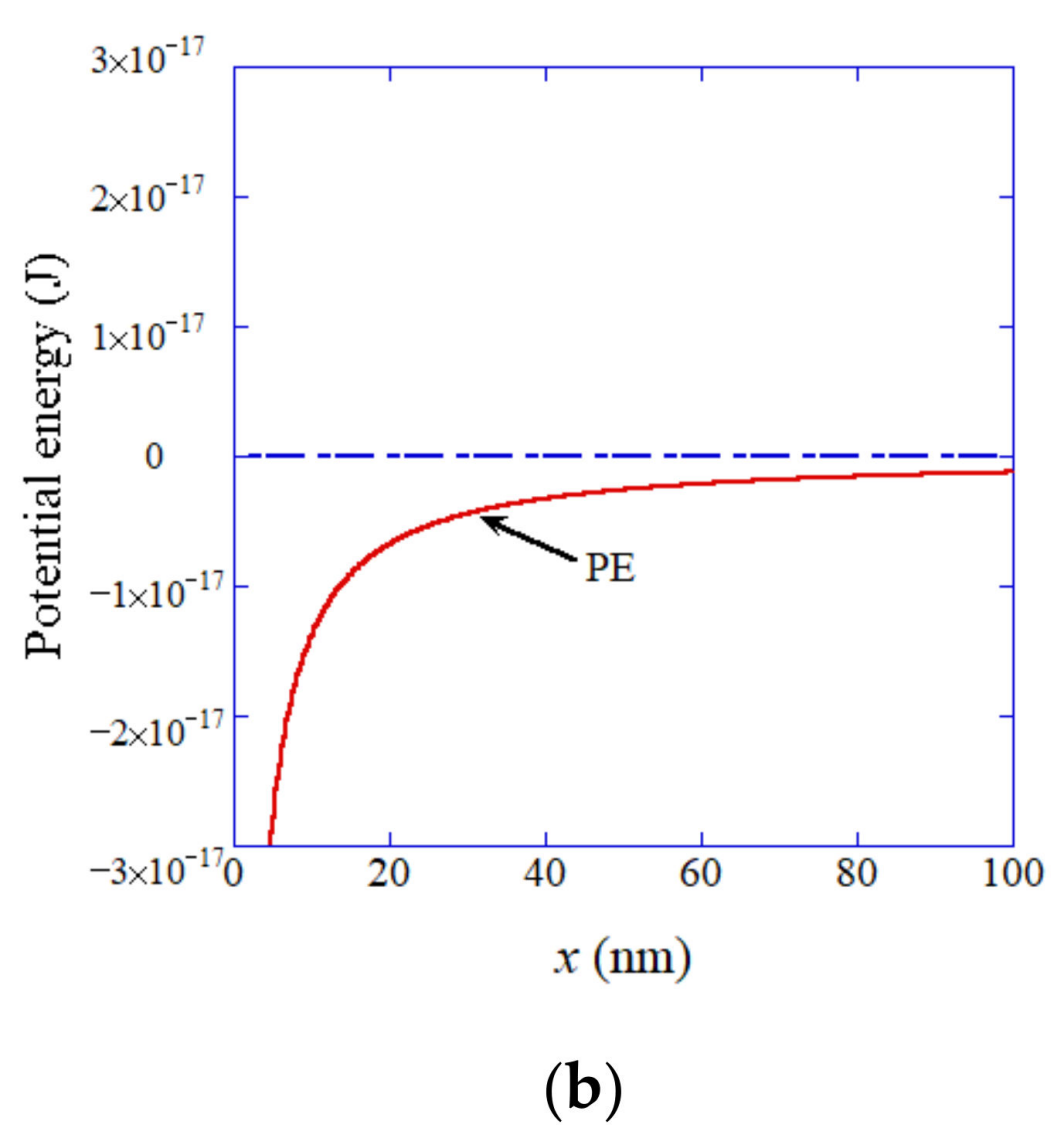
Total potential between a solid surface and a UFB as a function of the distance (x) between their surfaces [127]. (**a**) Glass, nylon ball, and PET. (**b**) PE. Copyright (2021), with permission from American Chemical Society.

According to the dynamic equilibrium model [29] of UFBs discussed in Section 2, more than half of the surface of a UFB is covered with a hydrophobic material. Accordingly, there is a hydrophobic attraction between a polymer surface and the hydrophobic material covering a UFB (Figure 12) [127]. In order to calculate the adsorption rate of UFBs on a polymer surface, the following interaction potentials are numerically calculated between each polymer material and the hydrophobic material covering a UFB: hydrophobic interaction, electric double-layer interaction, and van der Waals interaction. The calculated total potential is shown for each polymer material as well as glass in Figure 13 [127]. From the calculated total potential, the height of the potential barrier is obtained, which is used in the calculation of the adsorption rate. The potential barrier appears because attractive hydrophobic interaction is dominant near a solid (polymer) surface and the repulsive electric double-layer interaction is dominant apart from a solid surface. For PE in Figure 13b, the height of the potential barrier is zero because the attractive hydrophobic interaction is always dominant. The role of van der Waals interaction is minor, at least for the polymer materials studied here. The height of the potential barrier for a nylon ball is larger than that of PET because a nylon ball is hydrophilic, while PET and PE are hydrophobic.

According to the numerical calculations of the adsorption rate of UFBs on each polymer surface, the estimated time for adsorption is several orders of magnitude shorter than the experimental results of about 10 days for PET and PE [127]. It is a surprising result because normally, the theoretically estimated adsorption time is longer than the experimental data when the electric double-layer interaction is repulsive, as inhomogeneous distribution of electric charges on an actual solid surface is not taken into account in the theory [128]. The actual distribution of surface charges is as follows [128,129]. Only patch-like regions are charged, and the other areas do not contribute to the charge on the surface. Thus, the actual repulsive double-layer interaction is weaker than the theoretical estimates in which inhomogeneous distribution of charges is neglected. This picture is valid when the charging mechanism is due to $-OH^{-}$ groups on the surface, such as in the case of metal oxides and polymers [57,128,129]. For an ionic crystal surface, on the other hand, inhomogeneous distribution of charges results in stronger double-layer interaction [130].

With regard to nylon balls, theoretically estimated time for adsorption is considerably longer than the experimental data, which is in agreement with the usual tendency of theoretical estimates. Adsorption of UFBs on the glass wall of the container is negligible according to the present theoretical estimates.

**Figure 14:**
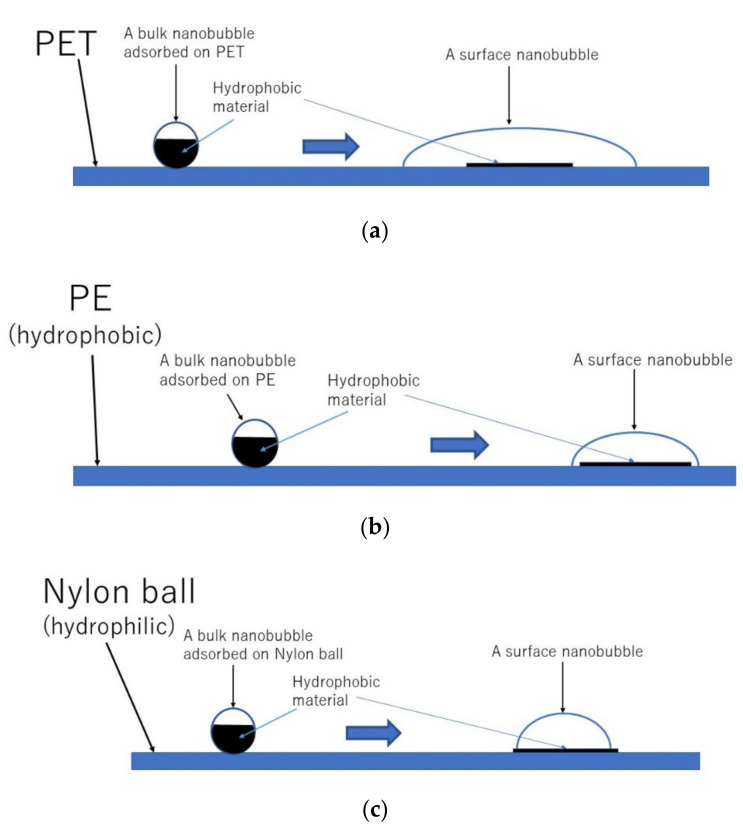
Proposed hypothesis [127]. (**a**) PET, (**b**) PE, and (**c**) nylon ball. Copyright (2021), with permission from American Chemical Society.

There is another mystery in the experimental results that the surface concentration of UFBs on PET and PE was more than an order of magnitude lower than the typical value of $10^{9}$ cm^−2^ for the colloid particles of similar or larger size [131]. To solve the two mysteries, it is proposed that UFBs change to surface nanobubbles on a hydrophobic surface with a footprint diameter of about 1 μm because surface nanobubbles block UFBs from adsorbing on a solid surface (Figure 12 and Figure 14) [127]. As surface nanobubbles block UFBs from further adsorption on a solid surface, the actual adsorption rate considerably drops below the theoretical estimates (the solution for the first mystery). As the footprint diameter of a surface nanobubble of about 1 μm is much larger than that of a spherical UFB of about 70 nm, the number concentration of UFBs, which change to surface nanobubbles, on a hydrophobic surface (PET and PE) is more than an order of magnitude lower than the typical value of $10^{9}$ cm^−2^ for the colloid particles of similar or larger size (the solution for the second mystery).

According to the experimental results [3], the surface concentration of UFBs on PET is about one order of magnitude smaller than that on PE. It may be due to larger surface nanobubbles on PET than those on PE (Figure 14a,b). The footprint diameter of a surface nanobubble may be determined by the surface concentration of pinning sites, which are impurities and tiny scratches on a solid surface [44,47]. Thus, the surface concentration of pinning sites on PET may be lower than that on PE used in the experiment.

As a surface nanobubble is usually unstable on a hydrophilic surface [44,47], the footprint diameter of a surface nanobubble on a nylon ball may be limited by the surface area of the hydrophobic material attached to a solid surface from the surface of the UFB (Figure 14c). In many other cases, surface nanobubbles on a nylon ball may completely disappear due to the hydrophilic nature of the surface. Accordingly, for the case of nylon balls, UFBs are not blocked from adsorbing on a solid surface, which may result in the normal adsorption of UFBs on a solid surface. In conclusion, on a hydrophobic surface (PET and PE), UFBs may change to surface nanobubbles with their footprint diameter of about 1 μm. The hypothesis should be checked by experimental observation of surface nanobubbles on a hydrophobic surface in UFB water in future.

## 6. Conclusions

The models for the stability of a UFB against dissolution were reviewed, such as the charge-stabilization model and high inner-density model. It is suggested that the dynamic equilibrium model is promising because there is TEM observation, as well as the fact that the reduction in “surface tension” of UFB water could be explained by the model; more than half of the surface of a UFB is covered with a hydrophobic material. The production of OH radicals is mostly during hydrodynamic or acoustic cavitation to produce UFBs. After ceasing cavitation, OH radicals may be produced from the chemical reaction of H_2_O_2_ and O_3,_ which are generated during cavitation. UFBs are concentrated on the liquid surface with their covered surface directed above the liquid surface. Such UFBs accelerate the rupture of the liquid film, which may result in the smaller value of “surface tension” measured by the du Noüy ring method. It is suggested that UFBs change to surface nanobubbles on a hydrophobic surface, which block UFBs from further adsorption on the solid surface. 

## Data Availability

Not applicable.
